# Freezing of Oocytes and Its Effect on the Displacement of the Meiotic Spindle: Short Communication

**DOI:** 10.1100/2012/785421

**Published:** 2012-05-02

**Authors:** János Konc, Katalin Kanyo, Rita Kriston, József Zeke, Sándor Cseh

**Affiliations:** ^1^Infertility and IVF Center of Buda, Szent Janos Hospital, Budapest 1125, Hungary; ^2^Faculty of Veterinary Science, Szent István University, István u. 2, Budapest 1078, Hungary; ^3^Subsidized Research Unit, Hungarian Academy of Sciences, 1525 Budapest, Hungary

## Abstract

Our investigations focused on spindle dynamics/displacement in frozen-thawed human oocytes. In each oocyte, prior to freezing and after thawing and culturing, the presence/location of the spindle was determined with the Polscope technique. A total of 259 oocytes have been thawed with a survival rate of 81.1%. From the 210 survived oocytes, 165 were fertilized (78.6%) and 89.1% of them cleaved. A total of 143 embryos were transferred into 63 patients resulting in 11 clinical pregnancies (17.5%), 7 of which resulted in live birth of 8 babies (1 twin pregnancy). We were able to detect the spindle in 221 of 259 oocytes (85.3%). After thawing and culturing the oocytes, we were able to visualize the spindle in 177 of 210 oocytes (84.3%). In 83 of these 177 oocytes, the spindle was observed to be in the same location as it was before cryopreservation (46.9%). However, in 94 of these 177 oocytes (53.1%), the spindle reformed in a different position/location relative to the polar body. Our results show that after thawing and culture in half of the spindle-positive oocytes the spindle was detected in a new location, indicating that the spindle and the polar body move relative to each other.

## 1. Introduction

In MII oocytes, the meiotic spindle is a temporary and dynamic structure of microtubules that depends on the process of tubulin polymerization and depolymerization. The spindle is crucial for the events following fertilization in the completion of meiosis, second polar body formation, migration of the pronuclei, and formation of the first mitotic spindle. The damage and/or absence of the spindle compromise the ability of the oocyte to fertilize and undergo normal preimplantation development [1]. As a sensitive structure, the meiotic spindle may be affected by the temperature decrease and physical stresses occurring during cooling, dehydration/rehydration, and the phase transitions of extracellular water during cryopreservation [2, 3]. Therefore, cooling and cryopreservation cause the meiotic spindle to undergo depolymerization. Spindle reformation/repolymerization and function play an important role in the further development of the oocyte following cryopreservation. Recently obtained data show that the sensitive, but highly dynamic microtubular structure of the spindle can be repolymerized with the return of physiological conditions and the majority of them have a normal barrel-shaped spindle with chromosomes aligned [1, 4–6].

Numerous studies on the effect of cooling/freezing on spindle morphology have been reported; however, to the best of our knowledge, no data is available connected with the possible displacement of the reformed/reappeared meiotic spindle in thawed human oocyte. Our investigations focused on spindle dynamics/displacement in frozen-thawed human oocytes. In each oocyte, prior to freezing and after thawing and 3 hours in vitro culture—just prior to ICSI—the presence and location of the spindle was determined with the spindle view technique of Polscope.

## 2. Materials and Methods

For spindle examination, each oocyte was placed in a 5 *μ*L drop of G-MOPS medium (G-MOPS, Vitrolife, Sweden) covered with oil (Ovoil, Vitrolife, Sweden). The system is composed of a temperature controller, a stage adapter, and the Delta T.C.O. dish with a specially coated glass bottom (Willco-dish, Willco Wells, The Netherlands). Oocytes were imaged by a Nikon Diaphot microscope with a video camera, objective lens, and controller, combined with a computerized imaging analyses system. In each oocyte, the presence and location of the spindle relative to the polar body positioned to 6 o'clock (holding pipette was positioned to 9 o'clock relative to the polar body) was detected and recorded (Spindle View Imaging System, CRI, Great Britain).

Mature human oocytes were frozen with slow protocol. Equilibration was performed at room temperature in freezing medium based on PBS + 20% HAS and containing 1.5 M propylene-glycol (PrOH) + 0.3 M sucrose. Oocytes were frozen in straws (max. 3 oocytes per straw) in Planer III Kryo 10 cell freezer (Planer Products Ltd., Sunbury-on-Thames, UK). After seeding at minus 6°C, oocytes were slowly cooled (−0.3°C/min) to −30°C, then they were cooled at a higher speed (−50°C/min) to –150°C before plunging into liquid nitrogen. Thawing was carried out by warming straws at room temperature for 30 s, followed by immersion in a 30°C water bath for 40 s. After thawing, oocytes were rehydrated and PrOH was removed from the eggs in four steps by passage through mediums based on PBS + 20% HAS supplemented with 0.5 and 0.3 M sucrose.

Before freezing and after thawing prior to ICSI in all oocyte, the spindle was visualized using the Polscope technique. In vitro fertilization with ICSI was performed after thawing and 3 hours in vitro culture. Fertilization was assessed 12–16 hours later. Embryo transfer was carried out 48–72 hours after ICSI at the 2 to 8 cell stages. Clinical pregnancy was defined by the presence of an intrauterine gestational sac and fetal heart beat on an ultrasound performed at 7 weeks of gestation.

## 3. Results

In the frame of this study, a total of 259 eggs have been thawed with a postthaw survival rate of 81.1% (210/259). From the 210 survived oocytes 165 fertilized (165/210; 78.6%) and out of the 165 fertilized oocytes 89.1% (147/165) cleaved. A total of 143 embryos were transferred into 63 patients (2.2 embryos per patient) resulting in 11 clinical pregnancies (11/63; 17.5%), 7 of which resulted in live birth of 8 babies (1 twin pregnancy). Four patients experienced spontaneous abortion between 8 to 12 weeks of pregnancy. The implantation rate per transferred embryo was 8.4% (12/143) and 4.6% (12/259) per egg thawed (Table 1). Chorion biopsy or amniocentesis was performed in all cases, and no chromosomal anomalies were observed.

**Figure 1:**
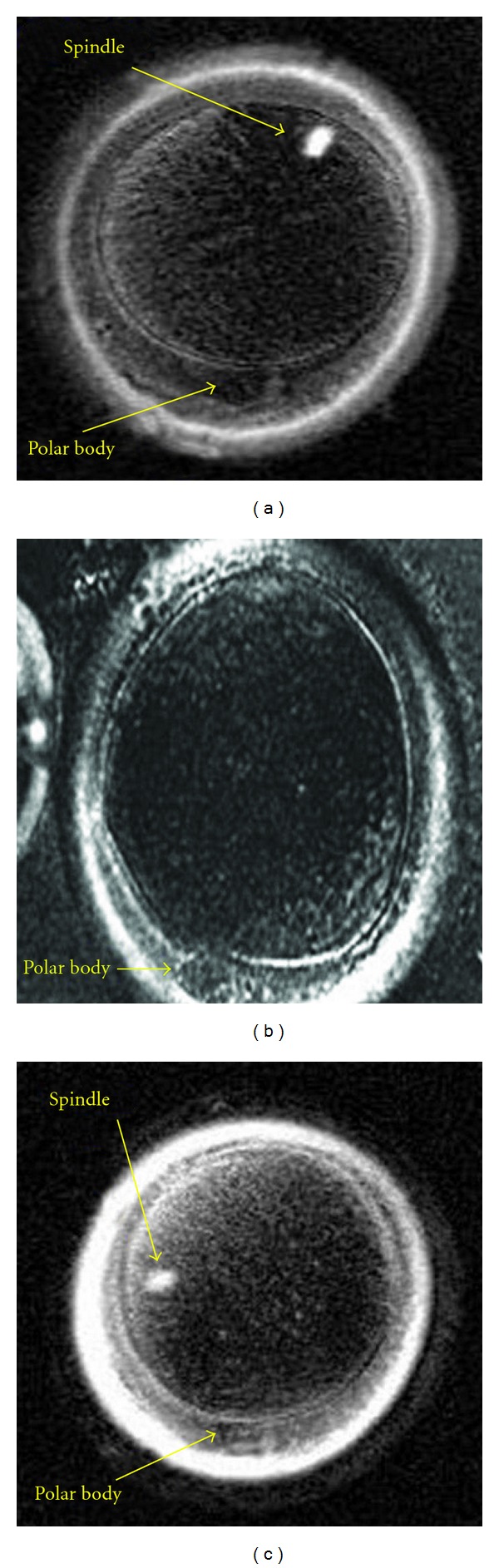
The sequence of the three figures of the same oocyte illustrates that the spindle and the polar body move relative to each other.

We made similar observations for a total of 259 oocytes and were able to detect the meiotic spindle in 221 (221/259; 85.3%) of them. After thawing and culturing the oocytes, we were able to visualize the spindle in 177 of 210 oocytes (177/210; 84.3%). In 83 of these 177 oocytes, the spindle was observed to be in the same location as it was before cryopreservation (83/177; 46.9%). However, in 94 of these 177 oocytes (94/177; 53.1%), the spindle reformed in a different position or location relative to the polar body, as illustrated in Figures 1(a), 1(b), and 1(c) (Table 2).

## 4. Discussion

It has been well documented that cooling and cryopreservation cause the meiotic spindle to undergo depolymerization [1–3, 6, 7]. The spindle is very sensitive to low temperature and cryoprotectants; however, the changes and recovery of it from cryopreservation have been linked to the function of oocytes in the process of fertilization and early embryo development [4, 6, 7]. Oocytes analyzed immediately after thawing displayed severe disorganization or disappearance of spindle using slow or vitrification methods. However, culturing oocytes for 1 to 3 hours after cryopreservation allows the spindle to repolymerize [4–8]. The degree and speed of recovery of the spindle depend on time intervals after thawing and on the freezing method. Ciotti et al. [9] reported that spindle recovery was faster in vitrification than in slow freezing [8], although Cobo [4] found comparable spindle recovery from vitrification and slow freezing after 3 hours of incubation [4].

Our result obtained with the noninvasive spindle view technique of Polscope supports the observation of others' that although the spindle transiently disappears immediately after thawing, it reorganizes/reforms with good morphology after 1–3 hours of in vitro culture at 37-38°C in the majority of mature oocytes [1, 6–8]. Our results indicate that by observing the response of individual oocytes the spindle does not always reform in its original position within the oocyte.

In conclusion, to the best of our knowledge, no data have been published yet concerning the possible displacement of the reformed/reorganized spindle inside the oocyte. Our results show that after thawing and culture in ~half of the oocytes in which the spindle was rebuilt/visualized it was detected in a new location and not at the initial place, indicating that the spindle and the polar body move relative to each other. Our observation provides further evidence of that using recently developed cryopreservation protocols both the capability of meiotic spindle for repolymerization and the original microtubule structure of the spindle can be maintained in high degrees.

## Figures and Tables

**Table 1:** Overall clinical results of frozen oocyte cycles.

No. of transferred cycles	63
No. of transferred embryos	143
No. of clinical pregnancies (%)	11 (17.5)
No. of live births	8*
No. of abortions (%)	4 (36.3)
Implanted rate per transferred embryos (%)	8.4
Implantation rate per thawed oocytes (%)	4.6

*1 twin pregnancy.

**Table 2:** Overall results of spindle dynamic analyses.

No. of thawed oocytes	259
No. of oocytes with spindle prior to freezing (%)	221 (85.3)
No. of oocytes with spindle after thawing (%)	177 (84.2)
No. of thawed oocytes with displaced spindle (%)	94 (53.1)
No. of survived and inseminated oocytes (%)	210 (81.1)
No. of fertilized oocytes (%)	165 (78.6)
No. of cleaved oocytes (%)	147 (89.1)
No. of embryos (%)	143 (97.3)
